# 

**Published:** 1902-04

**Authors:** 


### CONTENTS.

ORIGINAL ARTICLES:

Anthrax in a Horse. By W. HAMILTON, V.S. . . . . . . . . . . 201

Chronic Atrophic Orchitis in a Bull. By H. C. SIMPSON, D.V.S. . . . . . . 203

Urethral Calculus. By E. G. MARTEN, M.D.C. . . . . . . . . . 205

A Cow Case. By G. P. STATTER, V.S., M.D.V. . . . . . . . . . 207

Open Joint. By J. THOMSEN, V.S. . . . . . . . . . . 208

Typhoid Fever in a Horse. By L. U. SHIPLEY, D.V.S. . . . . . . . . 210

Parturition Cases. By WILLIAM DRINKWATER, V.S. . . . . . . . 21[illegible]

Rabies. By D. E. BAUGHMAN, M.D.C. . . . . . . . . . . 214

A Trip to South Africa. By J. MASSIE, Veterinary Major . . . . . . 221

President's Address. By S. J. J. HARGER, V.M.D. . . . . . . . . 224

Practical Hints in Pathology. By D KING SMITH, M.D. . . . . . . 228

The Unsolved Problems of Milk Fever. By J. M. W. KITCHEN, M.D. . . . . 230

DEPARTMENT OF CANINE, FELINE, AND AVIAN MEDICINE AND SURGERY . 235

ABSTRACTS FROM FOREIGN JOURNALS . . . . . . . . . . . . 237

REPORTS OF CASES:

An Abnormal Fœtus. By J. H. LUFF, PH.G., V.S. . . . . . . . . 240

Actinomycosis in a Dog. By H. W. GOHN, V.S. . . . . . . . . . 241

Poisoning by Feeding on Sinapis Nigra. By F. J. ROUB, V.S . . . . . 242

EDITORIALS . . . . . . . . . . . . . . . . . . 245

A. V. M. A.—NECROLOGY—SOCIETY NOTES—CORRESPONDENCE—SOCIETY PROCEEDINGS—PERSONALS—VETERINARY REGISTER (*continued*) . . . 247–267

